# 1st Symposium on Polydopamine and NanoTech Poland 2018: Conference Report

**DOI:** 10.3390/biomimetics3040037

**Published:** 2018-11-27

**Authors:** Radosław Mrówczyński, Marco d’Ischia, Haeshin Lee, Stefan Jurga

**Affiliations:** 1 NanoBioMedical Centre, Adam Mickiewicz University, Umultowska 85, 61-614 Poznań, Poland; stjurga@amu.edu.pl; 2 Department of Chemical Sciences, University of Naples Federico II, Via Cintia 4, I-80126 Naples, Italy; dischia@unina.it; 3 Department of Chemistry, Korea Advanced Institute of Science and Technology (KAIST), 291 University Road, Yuseong-gu, Daejeon 34141, South Korea; haeshin@kaist.ac.kr

**Keywords:** nanotechnology, nanomaterials, nanostructures, polydopamine, catechols, bioinspired materials

## Abstract

The NanoTech Poland is an annual international conference with a strong scientific agenda focused on nanotechnology in energy, environment, and biomedicine. The Nanotech Poland 2018 was held at the NanoBioMedical Centre and Department of Physics at Adam Mickiewicz University in Poznań from June 6th to June 9th. The aim of NanoTech Poland 2018 was to bring together the scientific community’s principal investigators, scientists, researchers, analysts, clinicians, policy makers, industry experts, and well-established and budding entrepreneurs to discuss the present and future perspectives in nanotechnology and nanoscience research and development. This year, the 1st Symposium on Polydopamine was held on June 6th. This forum was dedicated to the application of polydopamine and related catechol materials in a variety of research fields, both at the nano- and macroscale. The symposium gathered leading scientists from this important research field from top universities and institutions that have been involved in the research revolved around polydopamine. With over 200 national and international participants, NanoTech Poland 2018 and the 1st Symposium on Polydopamine provided a forum to present and discuss the latest scientific news from the field of nanotechnology with a strong interdisciplinary aspect and bioinspired materials.

## 1. Introduction

The NanoTech Poland 2018 international conference served as a forum dedicated to a multidisciplinary approach toward nanotechnology, where biologists, chemists, physicists, material scientists, and engineers found an opportunity to share their ideas, knowledge, and latest achievements in the field. The conference was organized by the NanoBioMedical Centre in collaboration with the Faculty of Physics at the Adam Mickiewicz University from June 6th to June 9th_,_ 2018. Both units are located at the campus of the Adam Mickiewicz University in Poznań near the beautiful wilderness. The NanoTech Poland originally started as a small workshop entitled “Summer Symposium on Nanomaterials and their application to Biology and Medicine” in 2011. After four meetings held in Poznań, the series grew and became NanoTech Poland in 2016. From that moment, this conference has been a gathering of leading scientists and exhibitors working in the field of nanotechnology. From the very beginning, this event was organized by Prof. Stefan Jurga with the strong support of an international advisory board and scientific committee.

The NanoTech Poland conference series has grown beyond its initial scope, and this year, the 1st Symposium on Polydopamine took place during the conference. It was the first time that such an event had been organized, and it is a new initiative which was supposed to assure the international forum dedicated to the application of polydopamine and related catechol materials in a variety of research fields, both at the nano- and macroscale. The 1st Symposium on Polydopamine gathered outstanding scientists who perform research revolved around polydopamine and related catecholic materials in the area of materials chemistry, nanomedicine, agriculture, and chemical physics. The symposium gave a chance to the international community to discuss the current situation in the field of catechol-based materials and to propose new directions and initiatives in this emerging area. 

The integral element of the 1st Symposium on Polydopamine and NanoTech Poland 2018 was the Exhibition, which provided a place for valuable networking and interactions between science and the industry. The Exhibition was targeted to entrepreneurs, scientists and companies, which directly or indirectly deal with nanoscience and nanotechnology. 

This year, the conference had four gold sponsors: InnoTherapy (http://www.innotherapy.com), Renishaw (http://www.renishaw.pl), PikInstruments (http://www.pik-instruments.pl/), and Tescan (http://www.tescan.com). InnoTherapy is an emerging company that produces hemostatic materials based on bioinspired catholic materials, and particularly supported the 1st Symposium on Polydopamine. Renishaw is a worldwide known company that develops devices for precise measurements and medical systems. PikInstruments is a leading Polish company that distributes advanced laboratory equipment and provides electron microscopy services. Tescan manufactures scientific instruments and laboratory equipment, including scanning electron microscopes, supplementary accessories for scanning electron microscopes, and light optical microscopy accessories and image processing. Other exhibitors like Jeol and Uni-Expert also supported the conference. The book prizes were awarded by ABE IPS. The media patronage was assured by laboratory.net, Nanonet, Laboratorium, Brec, biomat.net, and the journal *Biomimetics*, which particularly supported the 1st Symposium on Polydopamine. 

Moreover, the conference organizers recognized early-stage researchers, and therefore, cordially invited young researchers to present their recent scientific accomplishments in a short oral presentation during the Young Researchers Forum.

It is worth highlighting that the conference was held under the auspices of His Magnificence Rector of the Adam Mickiewicz University in Poznań, Prof. Andrzej Lesicki.

## 2. 1st Symposium on Polydopamine

The Symposium was organized into eight invited lectures and one sponsor lecture. All lectures were given by internationally well-known experts in the field of polydopamine. The forum was opened by the main organizer, Dr. Radosław Mrówczyński (NanoBioMedical Centre, Adam Mickiewicz University in Poznań, Poland), who also chaired the first session of the day. 

In the first talk, Prof. Haeshin Lee (Korea Advanced Institute of Science and Technology (KAIST), South Korea), pioneer and founder of the polydopamine field, summarized the last decade of the application of polydopamine and related catechol in different research areas, with an emphasis on new polymerization methods of polydopamine, its structural variability, and possible medical applications [1,2,3,4].

In the next presentation, Prof. Vincent Ball (Institut National de la Santé et de la Recherche Médicale, France) demonstrated size-controlled and colloidally stable polydopamine-based nanoparticles that were synthesized in acidic conditions, where autoxidation of dopamine was suppressed, using sodium periodate as the oxidant and a protein-like alkaline phosphatase as a templating agent. The size of the obtained nanoparticles was dependent on the dopamine/enzyme ratio, and the obtained particles displayed the enzymatic activity of alkaline phosphatase, with activity extending up to two weeks after the synthesis of particles. He also pointed out the cation interaction of tailored peptides and its influence on polymerization of the process of polydopamine [5,6,7].

Prof. Marco d’Ischia (University of Naples Federico II, Italy) gave an exhaustive lecture revising the current knowledge regarding the formation of polydopamine films. He emphasized in his lecture the role of catechol and amine in the adhesion process. He also showed the results of his work on understanding the formation of the polydopamine film in the presence of different diamines using various polymerization conditions, like simple autoxidation and sodium periodate [8,9,10,11]. 

The next session was chaired by Prof. Haeshin Lee, and the first speaker was Dr. Daniel Ruiz-Molina (Catalan Institute of Nanoscience and Nanotechnology (ICN2), Spain) who presented the application of ammonia-triggered polymerization of catechol derivatives for the preparation of hydrophobic coatings, which present the ability to provide robust and efficient water repellency on weaved textiles, including hydrophilic cotton. Further, he showed that mesoporous silica particles coated afterward can serve as an active template in preparation for polydopamine nanocapsules. Surprisingly, the core could be removed under an aqueous solution in mild conditions. In the end, he highlighted the usage of the bis-catecholic compound in preparation for new polymeric materials that could be successfully used as a gatekeeper for linked dyes inside the porous silica nanoparticles [12,13,14,15].

In the next speech delivered by Prof. Dr. Jürgen Liebscher (National Institute for Research and Development of Isotopic and Molecular Technologies (INCDTIM), Romania), new approaches toward analogues of polydopamine were presented. In his talk, he briefly summarized the state-of-the-art works in the field and showed existing routes toward the preparation of polydopamine analogues [16,17,18,19,20]. Later on, he focused on polymerization 3,4-dihydroxyhydrazide and its structural investigations. The data obtained from mass spectrometry (MS), solid-state nuclear magnetic resonance (ssNMR), and X-ray photoelectron spectroscopy (XPS) revealed the unexpected structure of the polymer where cycloaddition reactions were crucial steps in the formation of that material [21].



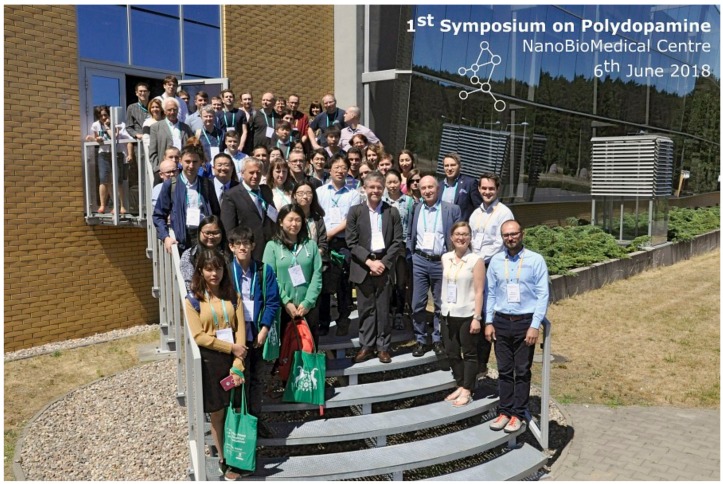

Picture of participants in the 1st Symposium on Polydopamine.

The last session of the Symposium was chaired by Dr. Daniel Ruiz-Molina. The session started with a talk by Dr. Radosław Mrówczyński who presented the application of polydopamine in preparation for multifunctional materials, both at the micro- and nanoscale, and addressed the main features of polydopamine responsible for its successful application in the field of nanomedicine. He also pointed out problems in liver cancer therapy and described a new material having contrasting properties in magnetic resonance imaging (MRI) assembling magnetic nanoparticles and polydopamine suitable for combined chemo- and photothermal therapy of liver cancer [22,23,24,25,26,27]. He then described the latest progress on the preparation polydopamine-based particles as a carrier for combined gene- and photothermal therapy of glioblastoma.

The application of polydopamine coatings for the sustained release of fertilizers and other agriculture ingredients was disclosed by Dr. Xin Jia (Shihezi University, China). He addressed the challenges in the application of nanomaterials to agriculture, and briefly presented the developments in this important field. He also showed new hollow materials from polydopamine obtained via polymerization of dopamine in EtOH in the presence of sodium chloride crystals, which served as a sacrificed template in the latter step. Such materials could be converted into carbon capsules after carbonization at high temperature under N_2_ atmosphere [28,29].

The Symposium’s last talk was delivered by Dr. Seonki Hong (Daegu Gyeongbuk Institute of Science and Technology (DGIST), South Korea). She focused on the structural investigation of polydopamine and its interaction with cations. She suggested that cation–π interactions between cations such as Na^+^, K^+^, NH_3_^+^, and electron-rich π systems (e.g., benzene, phenol, catechol, indole) could contribute to the self-assembly between dihydroxyindole-based progressive products and uncyclized amine functional groups constructing the polydopamine layer on a variety of surfaces in a material-independent manner [30,31].

Finally, a roundtable meeting took place where the current status of the field of polydopamine and related materials was discussed, and the new research opportunities were diagnosed. This meeting led to the establishment of new networks necessary for further improvements in the field of polydopamine and provided an opportunity to develop a long-term vision for the future development of catechol-based materials. 

The special poster session was organized in parallel to the planned poster session within the framework of the Nanotech Poland 2018. Seven posters were dedicated to this special Symposium, and the Best Poster Award was granted by the Poster Session Committee to Younseon Wang, who was also awarded by the journal *Biomimetics*.

## 3. Plenary Lectures at NanoTech Poland 2018

The NanoTech Poland 2018 was opened by Prof. Stefan Jurga, Director of the NanoBioMedical Centre, and the Rector of the Adam Mickiewicz University, Prof. Andrzej Lesicki. The conference had five invited plenary speakers, who were leading scientists in particular scientific areas.

The first lecture was given by Prof. Richard J. Spontak (North Carolina State University, NC, USA). He discussed the usage of functional thermoplastic elastomers, and the new ways to design new soft matter structures based on these materials. He also presented thermoplastic elastomers where the end blocks were mechanically uncompromised and capable of stabilizing a midblock-rich network in various environments. His final topic was about the selective sulfonation of polydiene and polystyrene midblocks in triblock copolymers, which are analogous to commercial midblock-sulfonated multiblock copolymers that can be successfully used in water desalination, gas separation, organic photovoltaics, and ionic polymer–metal composites [32,33,34].

Prof. Andrea Ferrari (University of Cambridge, UK) is the Director of the Cambridge Graphene Centre and the Engineering and Physical Sciences Research Council (EPSRC) Centre for Doctoral Training in Graphene Technology. He also serves as Science and Technology Officer and Chair of the the Graphene Flagship management panel. His talk was devoted to the third harmonic generation in two-dimensional (2D) nanomaterials and their application in photonics and optoelectronics [35]. 

Another plenary lecture was given by Prof. Jørgen Kjems (Aarhus University, Denmark). In his lecture, he demonstrated that the DNA origami technique allowed for specific external control of single enzyme activity by shielding the enzyme in a dynamic cage in order to gate its access to free substrate molecules. Furthermore, he explained that the hybridization of nucleic acids could also direct the assembly of multifunctional drug delivery devices in a manner called the “Lego brick”. In the next part of his lecture, he also presented the chemical modifications of obtained structures which allowed for the achievement of clinical relevance in vivo due to improved stability and pharmacokinetics. Thus, the successfully improved, targeted delivery of biologics to organs, tumors, and diseased tissue was revealed.

Prof. Silvia Marchesan (University of Trieste, Italy) showed how she took inspiration from d-amino acids and introduced them as short l-peptides to discover a wonderland of exciting nanostructures, and outlined their rules for assembly. Her lecture presented recent findings that have unveiled the molecular conformation(s) corresponding to a characteristic spectroscopic signature, and its evolution as a continuum from single molecules to the nanoscale and beyond, to macroscopic soft materials. Her group identified different nanostructures with varied nanomorphology and interesting features, such as the formation of water channels with different diameters [36,37,38,39,40].

The final lecture was given by Dr. Mario Rocca (University of Genoa, Italy). His talk was devoted to ultrathin MgO films, that are of special relevance because their energy gap is close to the bulk value already in the ultrathin limit, and therefore become an important case for further study. In his talk, he described the deposition of Ni on the MgO/Ag(100) ultrathin films [3] at a temperature of 200 K. Through his research, he found a dual growth mode giving rise to clusters of several tens of nanometers in diameter, and others consisting of just a few atoms. He also described further applications of MgO thin films [41,42,43].

## 4. Sessions

The NanoTech Poland 2018 was divided into two parallel sessions that were focused on nanobiomedicine and advanced nanomaterials. Overall, speakers gave 32 invited lectures and 14 contributed lectures within those two sessions. Detailed descriptions of each presented work lies outside the scope of this conference report; therefore, we strongly encourage those interested to go to the online version of the book of abstracts [44].



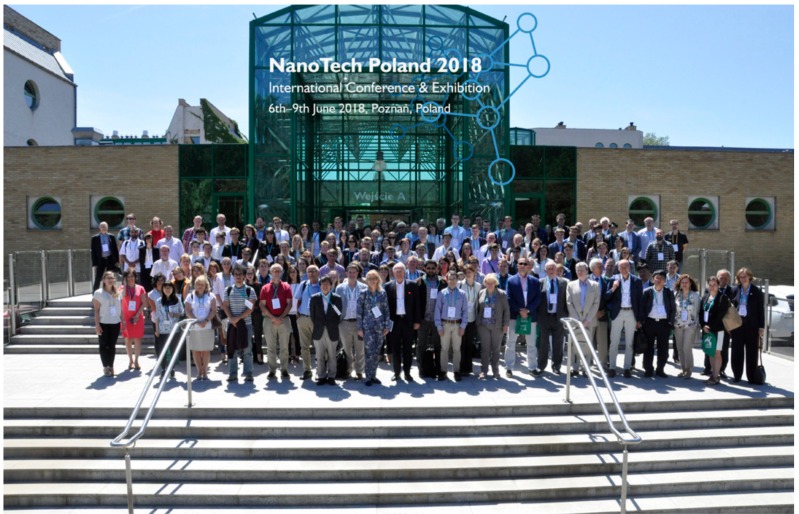

Picture of participants who attended the 1st Symposium on Polydopamine and NanoTech Poland 2018.

An integral part of the oral contributions was a poster session that attracted over 100 posters dedicated to different areas of nanotechnology and its application to the field of nanobiomedicine and materials preparation. Another key session during NanoTech Poland 2018 was the Young Researcher Forum, which gathered 24 scientists and allowed them to present their results obtained at an early stage in their scientific career.

The commission granted awards and honors to the following participants: Mieszko Kołodziej (Institute of Molecular Physics, Polish Academy of Sciences, Poland) for the Best Poster in Section A (advanced nanomaterials). The Distinction Poster Award was granted to Benjamin Jabłoński from the same institute. The Best Poster Award in Section B (nanobiomedicine) was given to Michał Wojasiński (Warsaw University of Technology, Poland). Furthermore, the Best Oral Presentation Award within the Young Researcher Forum was granted to Kaja Spilarewicz-Stanek (University of Lodz, Poland) in Section A; and to Krzysztof Żukowski (Adam Mickiewicz University, Poland) in Section B.

## 5. Conclusions

The 1st Symposium on Polydopamine and NanoTech Poland 2018 was a great success as a result of the high quality of the oral and poster contributions, as well as the warm and friendly atmosphere which helped strengthen existing networks and build new ones between scientists contributing efforts to the study of catechols and future of nanotechnology. It was also a great interdisciplinary forum to share ideas between scientists from different fields, and served to function as an effective venue where they could learn from one another. We are looking forward to the successful continuation of this conference and are pleased to announce that the NanoTech Poland 2019 will continue its annual cycle and will be held from the 5th to 7th June 2019. The location of the 2nd Symposium on Polydopamine will be moved to South Korea and organized in 2020, with Prof. Haeshin Lee as the Chair of the meeting. 
