# Erratum: 1st Symposium on Polydopamine and NanoTech Poland 2018: Conference Report. *Biomimetics* 2018, *3*, 37

**DOI:** 10.3390/biomimetics4010014

**Published:** 2019-02-07

**Authors:** Radosław Mrówczyński, Marco d’Ischia, Haeshin Lee, Stefan Jurga

**Affiliations:** 1 NanoBioMedical Centre, Adam Mickiewicz University, Umultowska 85, 61-614 Poznań, Poland; stjurga@amu.edu.pl; 2 Department of Chemical Sciences, University of Naples Federico II, Via Cintia 4, I-80126 Naples, Italy; dischia@unina.it; 3 Department of Chemistry, Korea Advanced Institute of Science and Technology (KAIST), 291 University Road, Yuseong-gu, Daejeon 34141, Korea; haeshin@kaist.ac.kr

The authors regret that few mistakes were made in the original publication by Mrówczyński et al. [1].

Within the Acknowledgments section, the paragraph
“The organizers would like to thank Ms. Dorat Flak, and Mrs. Lidia Szutkowska and Alicja Jorasz for their help in organizing the conference as well as for administrative support. We are grateful for all speakers and presenters for their valuable contributions to the conference, quality of their work, and their feedback in building a friendly atmosphere. The organizers also would like to thank the sponsors who provided support to the conference and brought valuable contribution.”
has been corrected to
“The authors would like to thank the entire organizing committee, in particular to Dr. Dorota Flak, Dr. Lidia Szutkowska, and Dr. Alicja Jorasz (NanoBioMedical Centre, Adam Mickiewicz University), for their invaluable commitment and effort. We are also grateful to Mr. Marek Nowak (Faculty of Physics, Adam Mickiewicz University), the author of the joint photo of the Symposium and NanoTech Poland participants.”

The authors would like to apologize for any inconvenience caused by these errors.
